# Site-Specific Integration and Expression of an Anti-Malarial Gene in Transgenic *Anopheles gambiae* Significantly Reduces *Plasmodium* Infections

**DOI:** 10.1371/journal.pone.0014587

**Published:** 2011-01-25

**Authors:** Janet M. Meredith, Sanjay Basu, Derric D. Nimmo, Isabelle Larget-Thiery, Emma L. Warr, Ann Underhill, Clare C. McArthur, Victoria Carter, Hilary Hurd, Catherine Bourgouin, Paul Eggleston

**Affiliations:** 1 Centre for Applied Entomology and Parasitology, Keele University, Keele, United Kingdom; 2 Department of Entomology, Virginia Polytechnic Institute and State University, Blacksburg, Virginia, United States of America; 3 Oxitec Ltd, Milton Park, Oxford, United Kingdom; 4 Institut Pasteur, Center for Production and Infection of Anopheles, Parasitology and Mycology Department, Paris, France; University of California Los Angeles, United States of America

## Abstract

Diseases transmitted by mosquitoes have a devastating impact on global health and this is worsening due to difficulties with existing control measures and climate change. Genetically modified mosquitoes that are refractory to disease transmission are seen as having great potential in the delivery of novel control strategies. Historically the genetic modification of insects has relied upon transposable elements which have many limitations despite their successful use. To circumvent these limitations the *Streptomyces* phage phiC31 integrase system has been successfully adapted for site-specific transgene integration in insects. Here, we present the first site-specific transformation of *Anopheles gambiae*, the principal vector of human malaria. Mosquitoes were initially engineered to incorporate the phiC31 targeting site at a defined genomic location. A second phase of genetic modification then achieved site-specific integration of Vida3, a synthetic anti-malarial gene. Expression of Vida3, specifically in the midgut of bloodfed females, offered consistent and significant protection against *Plasmodium yoelii nigeriensis*, reducing average parasite intensity by 85%. Similar protection was observed against *Plasmodium falciparum* in some experiments, although protection was inconsistent. In the fight against malaria, it is imperative to establish a broad repertoire of both anti-malarial effector genes and tissue-specific promoters for their expression, enabling those offering maximum effect with minimum fitness cost to be identified. In the future, this technology will allow effective comparisons and informed choices to be made, potentially leading to complete transmission blockade.

## Introduction

Despite intense efforts, malaria is responsible for almost one million deaths per year, predominantly in sub-Saharan Africa [1]. As a result of intervention, the mosquito vectors are increasingly resistant to pesticides [2] and the causative *Plasmodium* parasites are becoming resistant to widely used anti-malarial drugs [3]. In the absence of an effective vaccine, alternative strategies are badly needed. Control measures that focus on the vector remain the most effective and the deployment of transgenic mosquitoes, refractory to malaria transmission, is increasingly seen as having great potential [4]. In fact, some success has recently been reported in *Anopheles stephensi* [5]. The focus of this study is *Anopheles gambiae*, the major malaria vector in endemic regions of sub-Saharan Africa.

Insect transgenesis has relied upon transposable genetic elements which, despite their utility, have limited carrying capacity and their essentially random integration can cause insertional mutagenesis and position effects [6], [7]. The *Streptomyces* phiC31 site-specific transgene integration system circumvents these problems [8], [9] and can potentially accept much larger inserts than the 42 kb *Streptomyces* phage genome [10]. Site-specificity results from the two phase nature of the system. In phase 1, the phage attachment site (*attP*) is integrated into the genome using conventional transposon-mediated transgenesis. During phase 2 transformation, catalysed by integrase, the *attP* site accepts transgenes from plasmids containing the bacterial attachment site (*attB*). Integration recombines *attP* and *attB* sites into unique *attL* and *attR* sequences that are not recognised by the integrase, rendering insertions unidirectional and stable.

Adaptation of the phiC31 integration system for *Drosophila melanogaster* was successful [11] and we subsequently reported its use in the yellow fever and dengue virus vector, *Aedes aegypti* [9]. Here we report the first site-specific transformation of *An. gambiae* and its use to express a synthetic, anti-malarial effector gene, Vida3 [12], into the midgut lumen following a bloodmeal. The 14 amino acid Vida3 peptide, designed following a survey of peptides effective against complex outer membranes, was active against both early sporogonic stages and developing oocysts of murine malaria parasites in *An. gambiae* infections *in vivo* [12].

The results presented here demonstrate stable expression of Vida3 over a number of generations, which significantly reduced the intensity of *Plasmodium yoelii nigeriensis* infections. Whilst protection against the human parasite, *Plasmodium falciparum*, was inconsistent, a significant reduction in oocyst burden was observed under certain conditions. The expression of peptides screened against the human parasite, combined with site-specific transgene integration to allow direct comparisons to be made between potential effector genes, will provide valuable advances in the fight against malaria.

## Results

### Generation and characterisation of *attP* targeting strains

**Figure 1:**
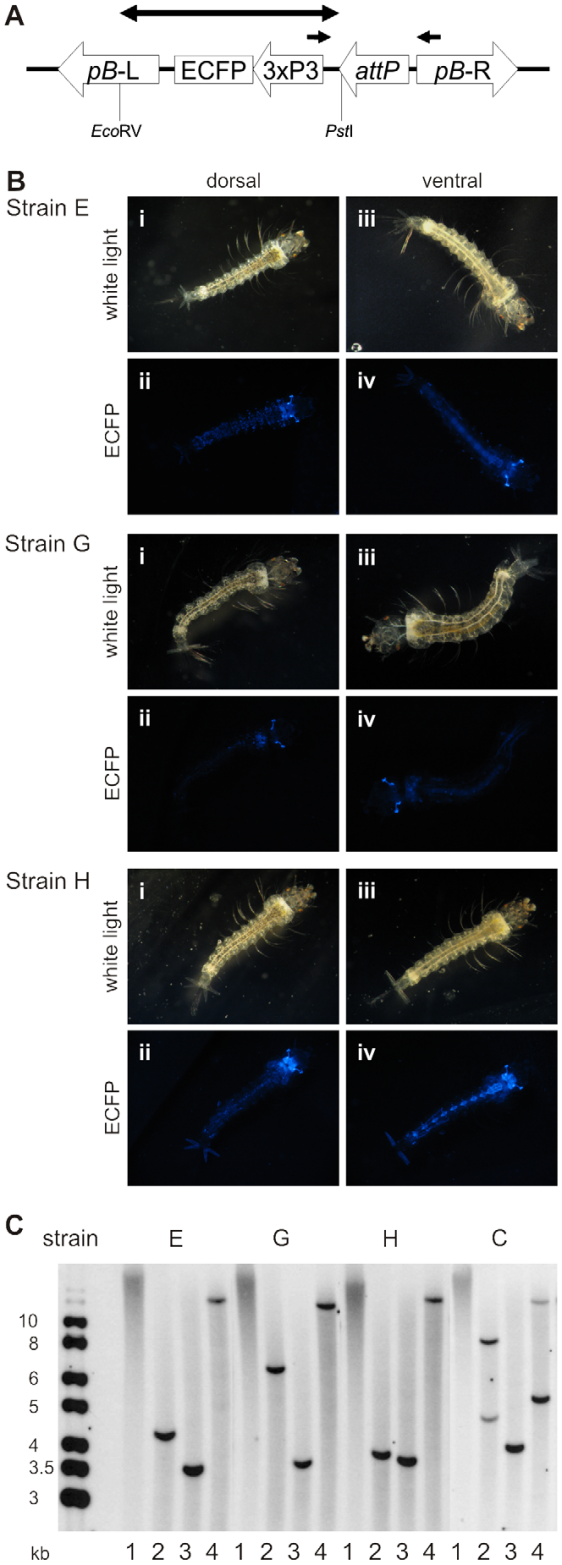
Generation and genomic analysis of *attP* targeting strains. (A) Transgene organisation of the *piggyBac* insertion from pBac[3xP3-ECFPaf]-attP (not to scale) showing an unoccupied *attP* target site, ECFP marker controlled by the *D. melanogaster* 3xP3 eye-specific promoter and *piggyBac* left and right terminal inverted repeats (*pB*-L and *pB*-R). The double headed arrow identifies the probe used in Southern blot analysis, excised using *Eco*RV and *Pst*I sites, and single arrows the location of PCR primers to amplify a 391 bp fragment spanning *attP*. (B) Fluorescence profiles of transgenic strain E, G and H larvae. For all strains: i, dorsal bright-field image; ii, dorsal ECFP image; iii, ventral bright-field image; iv, ventral ECFP image. In addition to eyes and optic nerves (all strains), fluorescence is visible in cerebral ganglia and anal papillae (strains E and H) and ventral nerve ganglia (strain H). (C) Southern blot analysis of phase 1 targeting strains E, G, H and C. Genomic DNA is either uncut (1) or digested with *Xmn*I (2), *Nsi*I (3) or *Pst*I (4) and probed with the 2195 bp *Eco*RV/*Pst*I fragment described above. The band generated following *Nsi*I digestion of strain C genomic DNA most likely represents a doublet.

Four independent *An. gambiae* targeting strains for phiC31 integrase were generated by *piggyBac*-mediated integration of the *attP* target site, linked to an eye-specific ECFP marker. In total, 2311 wild-type KIL strain embryos were injected with the phase 1 plasmid, pBac[3xP3-ECFPaf]-attP (Figure 1A) [9]. Surviving G_0_ adults were backcrossed to KIL and a number of G_1_ transformants identified. Four independent strains (C, E, G and H) were identified by unique patterns of ECFP expression in neural tissue (optic nerve, cerebral ganglia, ventral nerve ganglia and anal papillae), in addition to eye expression (Figure 1B).

Populations of targeting strains were established from single G_1_ positive adults by backcrossing to wild-type. Southern blot analysis identified single insertions in strains E, G and H and two inserts in strain C (Figure 1C). For each strain, sequence flanking one side of the genomic integration site was obtained from inverse PCR products. Alignment of sequences to the PEST genome using the Blast algorithm [13] allowed the other flanking sequence to be confirmed by genomic PCR using one primer within the transgene and a second designed to the adjacent PEST genome. All showed canonical *piggyBac* TTAA sequence duplication (Table S1), although the second integration site in strain C could not be resolved. The identified integration sites were mapped to chromosomes 3R (strains C and E) and 2L (strains G and H). The strain C insertion was located 2.5 kb from a predicted gene, whilst those for strains E, G and H were all more than 15 kb from predicted genes. Backcrosses confirmed normal Mendelian inheritance of the fluorescent marker in strains E, G and H (Table S2). No obvious fitness disturbances were apparent for any of the strains during normal husbandry and strain E was chosen for site-specific integration following enrichment for the targeting site.

### Expression of an anti-malarial effector gene by site-specific integration

**Figure 2:**
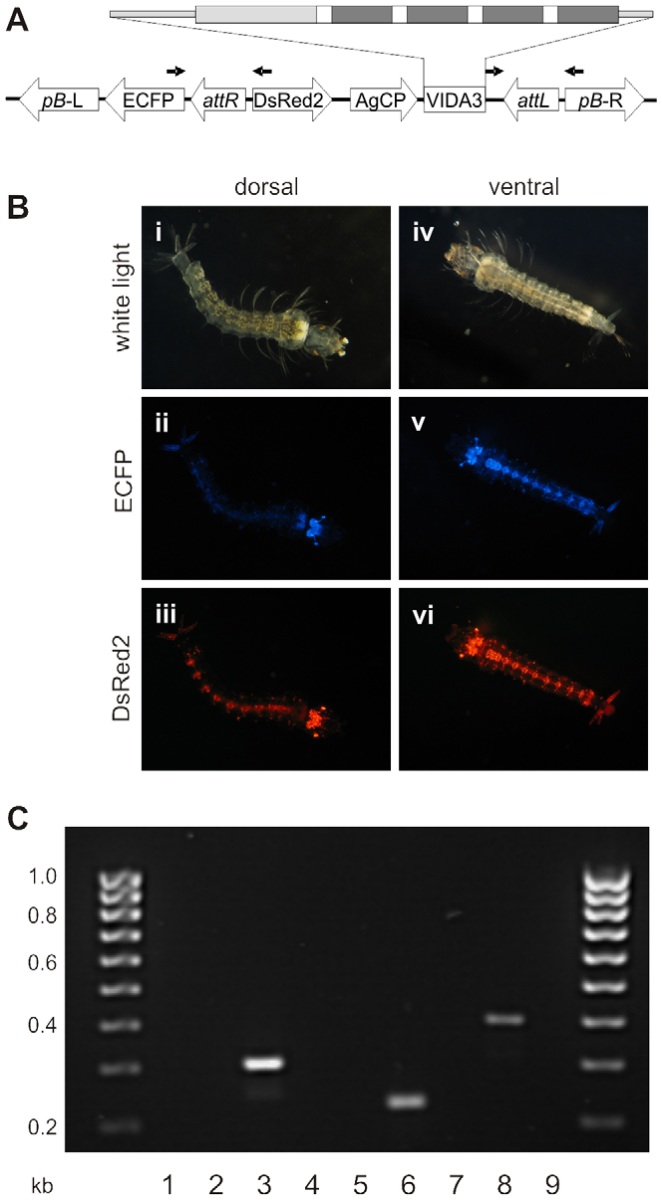
Genome organisation and characterisation of phase 2 site-specific integrations. (A) Organisation of the occupied target site (not to scale) following site-specific integration of pBattB-DsRed2-AgCP-vida tet and resolution of *attB* and *attP* into *attL* and *attR*. The insertion is flanked by phase 1-specific left and right *piggyBac* terminal inverted repeats (*pB*-L and *pB*-R) and the ECFP fluorescent marker. Vida3 is expressed from the A*n. gambiae* carboxypeptidase (*AgCP*) promoter and marked by 3xP3:DsRed2. The transcribed region (expanded) includes the *AgCP* 5′ and 3′ UTRs (light grey narrow boxes) and signal peptide (light grey wider box) and four copies of Vida3 (dark grey boxes), separated by linker sequences (white boxes). Arrows identify PCR primers that amplify fragments specific to *attL* and *attR*. (B) Fluorescence profiles of EVida3 phase 2 larvae; i, dorsal bright-field image; ii, dorsal ECFP image; iii, dorsal DsRed2 image; iv, ventral bright-field image; v, ventral ECFP image; vi, ventral DsRed2 image. In addition to eyes and optic nerves fluorescence is visible in cerebral ganglia, anal papillae and ventral nerve ganglia. Following integration in phase 2, strain E exhibits fluorescence in ventral nerve ganglia, which was not clearly visible in phase 1. This may reflect some degree of cross-talk between the two filter sets given the high intensity of the red fluorophore. Note also that DsRed2 expression is restricted to the cell nuclei by a nuclear localisation signal. (C) Confirmation of site-specific transgene integration in EVida3. PCR reactions with no template, strain E DNA or strain EVida3 DNA were established with specific primers for *attL* (301 bp, lanes 1, 2 and 3), *attR* (224 bp, lanes 4, 5 and 6) or *attP* (391 bp, lanes 7, 8 and 9).

Vida3, a synthetic anti-*Plasmodium* peptide [12], was chosen for expression in phase 2. The expression strategy previously used for the SM1 peptide was adopted to target early ookinetes in the ingested bloodmeal and ensure release of the very small peptide from ribosomes [14]. Vida3 was thus expressed as a tetramer using the *An. gambiae* carboxypeptidase (*AgCP*) promoter, signal peptide and untranslated regions (Figure 2A). In addition to an *attB* sequence, the phase 2 plasmid included an eye-specific DsRed2 fluorescence marker.

Phase 2 injections into 6124 strain E embryos generated two phase 2 transgenic larvae, one of which emerged as an adult male. The resulting EVida3 strain was identified by the expression of both ECFP and DsRed2 fluorescence (Figure 2B). Correct site-specific integration was confirmed by PCR across the resulting *attL* and *attR* regions in EVida3 (Figure 2C) and the sequence of cloned products confirmed. A backcross to wild-type confirmed normal Mendelian inheritance of both fluorescent markers in EVida3 (Table S2).

**Figure 3:**
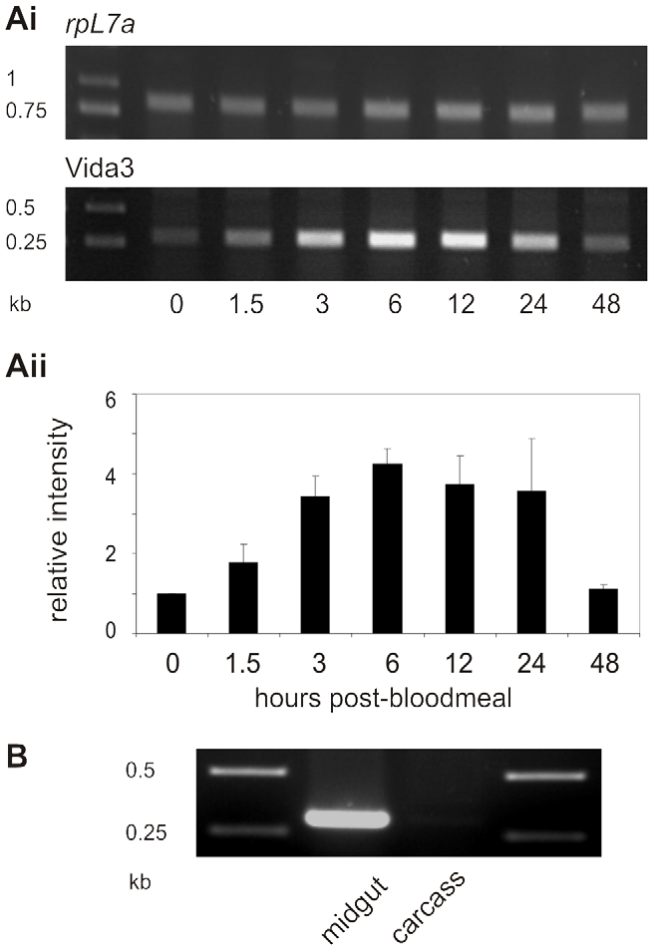
Expression of Vida3 from the *AgCP* promoter. (A) Expression of Vida3 in strain EVida3 following a bloodmeal. (i) Representative gels showing semi-quantitative RT-PCR products of *rpL7a* (15 cycles, upper panel) and Vida3 (25 cycles, lower panel) from samples taken at 0, 1.5, 3, 6, 12, 24 and 48 hours post-bloodmeal. (ii) Mean relative intensity with standard errors of Vida3 PCR products. Following quantification, Vida3 RT-PCR bands were normalised to ribosomal protein *rpL7a* RT-PCR products from the same samples and expressed relative to the 0 time point. The histogram represents data from a minimum of two replicates. (B) RT-PCR of Vida3 expression in the midgut and carcass 6 hours post blood-meal.

Semi-quantitative RT-PCR, on total RNA extracted from EVida3 females at different times post-bloodmeal, was used to determine the expression profile of the Vida3 tetramer from the *AgCP* promoter (Figure 3A). Intensity of PCR products, normalised to ribosomal protein L7a (*rpL7a*), showed upregulation from basal levels following a bloodmeal and peak expression after 6 hours. By 48 hours post-bloodmeal, mRNA abundance had returned to normal, thus expression is consistent with that reported for SM1 [14] and endogenous *AgCP* [15]. We confirmed exclusive expression of Vida3 in the midgut by RT-PCR on midgut and carcass 6 hours post-bloodmeal (Figure 3B). Vida3 expression in EVida3 has been stable for over 40 generations (data not shown).

### Infection challenge with Plasmodium yoelii nigeriensis

**Figure 4:**
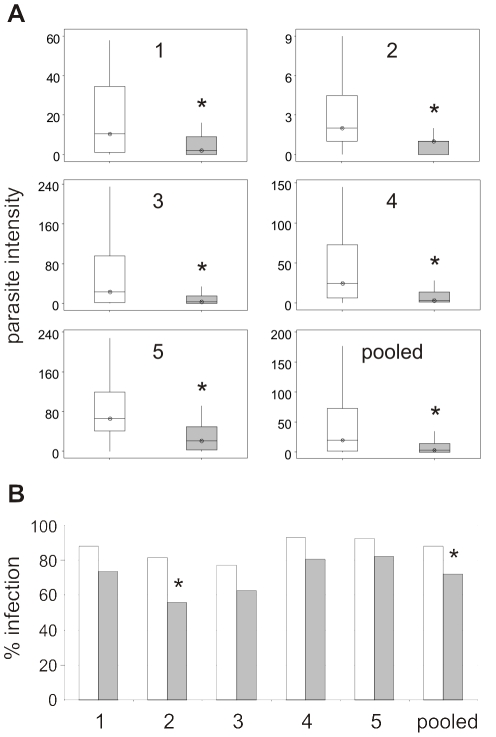
Parasite intensity and prevalence following *P. y. nigeriensis* infections of transgenic strains E and EVida3. (A) Boxplots show intensity for strains E (white boxes) and EVida3 (grey boxes) from five independent experiments and the pooled data. Oocysts and melanised ookinetes were scored as invading parasites. Vertical lines denote the 95% confidence interval, horizontal lines with symbol mark the median and the interquartile range of the data is boxed. Probabilities for significant reductions in intensity in EVida3 (*) in experiments are: 1, *P* = 0.008; 2, *P* = 0.0007; 3, *P* = 0.009; 4, *P*<0.0001; 5, *P*<0.0001 and pooled data 1–5, *P*<0.0001. (B) Histograms show prevalence for strains E (white bars) and EVida3 (grey bars) in experiments 1–5 and the pooled data. Results from individual experiments indicate a trend towards a lower prevalence of infection in EVida3, which is only significant (*) for experiment 2 (*P* = 0.02) and the pooled data 1–5 (*P*<0.001).

We observed no significant difference in female wing length, an accepted measure of body size, between strain E and EVida3 homozygotes (*P* = 0.127). Thus, we could infer that females from each strain would take similar sized bloodmeals during infection experiments. To test Vida3 activity against parasites, we first infected strains E and EVida3 with the murine parasite *Plasmodium yoelii nigeriensis*. Midgut infections were counted as a measure of intensity and prevalence. In addition to oocysts we frequently observed melanised ookinetes and, since these had traversed the midgut epithelium, they were included as invading parasites. Five independent infections were performed over two years (Table 1). Intensity of infection with *P. y. nigeriensis* was significantly reduced in EVida3, compared to E, in all five experiments. Since levels of infection varied considerably between experiments, data are shown as separate boxplots for independent experiments and the pooled data (Figure 4A) and as a composite plot in Figure S1. Experiments 1 to 5 resulted in decreased mean parasite burdens in EVida3 compared to E of 83%, 77%, 80%, 87% and 67% respectively. When the data from all five experiments are pooled, this reduction is very highly significant (*P*<0.0001). Although parasite burden varies significantly between experiments (*P*<0.0001) this interaction is not significant (*P* = 0.3). Prevalence of infection was also reduced in EVida3, although this reduction was only significant in experiment 2 (*P* = 0.02, Figure 4B).

**Table 1:** *P. y. nigeriensis* infections of transgenic strains E and EVida3.

	parasitaemia (exflagellation)	strain	n	median	interquartile range
1	7%	E	34	10.5	1–34.5
	(3)	EVida3	49	2	0–9
2	4%	E	33	2	1–4.5
	(1.7)	EVida3	36	1	0–1
3	8%	E	26	23	1.5–95.8
	(3)	EVida3	40	3	0–14.8
4	8%	E	44	24.5	6.25–72.75
	(1.9)	EVida3	56	3	1–13.5
5	9%	E	52	66	41.5–119.25
	(0.8)	EVida3	50	21.5	2.75–49.25
1–5		E	189	20	2–72.5
		EVida3	231	3	0–14

Details of mouse infections (parasitaemia and exflagellation), numbers of mosquitoes dissected (n), median and inter-quartile range of intensity of infections for experiments 1 to 5 and the pooled data.

### Infection challenge with *Plasmodium falciparum*

**Figure 5:**
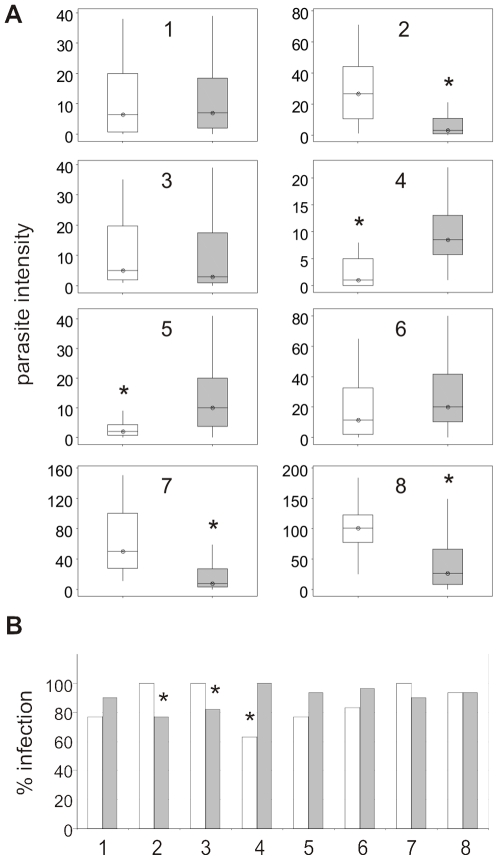
Parasite intensity and prevalence following *P. falciparum* infections of transgenic strains E and EVida3. (A) Boxplots show parasite intensity for strains E (white boxes) and EVida3 (grey boxes) from 8 independent experiments. Vertical lines denote the 95% confidence interval, horizontal lines with symbol mark the median and the interquartile range of the data is boxed. Significant reductions in intensity (*) were observed for experiments 2 (EVida3; *P*<0.0001), 4 (E; *P*<0.0001), 5 (E; *P* = 0.0002), 7 (EVida3; *P*<0.0001), 8 (EVida3; *P*<0.0001). (B) Histograms show prevalence for strains E (white bars) and EVida3 (grey bars) in experiments 1–8. Significant reductions (*) were observed for experiments 2 (EVida3; *P* = 0.01), 3 (EVida3; *P* = 0.03) and 4 (E; *P* = 0.0003).

To assess the impact of Vida3 on human parasites, E and EVida3 homozygotes were fed *in vitro* cultured *P. falciparum* gametocytes, in eight independent experiments, over a two year period (Table 2). Oocyst burden in independent experiments was found to be variable (Figure 5A). Significantly reduced parasite intensity in EVida3 compared to E was observed in experiments 2, 7 and 8 (*P*<0.0001 for all). In experiments 4 and 5, where control infections were lower, a significant increase in oocyst burden was observed in EVida3 (*P*<0.0001 and *P* = 0.0002 respectively). Analysis of the pooled data, which indicates a reduced burden in EVida3, does not show a significant difference to E (*P* = 0.13), although experiments are significantly different (*P*<0.0001), and have a significant interaction (*P*<0.0001). Since *P. falciparum* experimental infections are inherently variable, the oocyst burden was also analysed after excluding uninfected mosquitoes. Results were indicative of a reduced oocyst burden in EVida3 but statistically inconclusive (*P* = 0.05). For experiments 6 to 8, a second uninfected bloodmeal was given after three days to increase infections [16]. Analysis of pooled data from these experiments did identify a significant reduction in oocyst burden for EVida3 (*P*<0.0001), although variation between experiments remains a significant interaction (*P*<0.0001). Overall, when infections were high, expression of Vida3 did offer a significant level of protection. The prevalence of infection for pooled data showed no significant difference between E and EVida3, although differences were observed in individual experiments (Figure 5B). Prevalence in EVida3 was significantly reduced in experiments 2 (*P* = 0.011) and 3 (*P* = 0.025) but significantly increased in experiment 4 (*P* = 0.0003).

**Table 2:** Infection data for *P. falciparum* infections of strains E and EVida3.

	gametocytes (male ratio)	strain	n	median	interquartile range
1	3.2×10^7^	E	30	6.5	0.75–20
	(0.58)	EVida3	30	7	2–18.5
2	2.6×10^7^	E	30	26.5	10.5–44.25
	(0.60)	EVida3	30	3	0.75–10.75
3	1.9×10^7^	E	30	5	2–19.75
	(0.55)	EVida3	33	3	1–17.5
4	1.7×10^7^	E	30	1	0–5
	(0.77)	EVida3	30	8.5	5.75–13
5	2.3×10^7^	E	30	2	0.75–4.25
	(0.49)	EVida3	30	10	3.75–20
6	4.9×10^7^	E	30	11.5	2–32.5
	(0.56)	EVida3	30	20	10.25–41.5
7	3.3×10^7^	E	30	50	28–100.25
	(0.52)	EVida3	30	7.5	3–27.5
8	2.4×10^7^	E	30	101.5	77.25–122.75
	(0.60)	EVida3	30	26.5	8.5–66.5

Details of gametocyte infections, male ratio, numbers of mosquitoes dissected (n), median and inter-quartile range of intensity of infections for experiments 1 to 8.

## Discussion

In this study we have demonstrated the utility of *Streptomyces* phiC31 site-specific transgene integration as a tool that will help advance research capability in *An. gambiae*. We previously published the establishment of the phiC31 system in *Ae. aegypti* and demonstrated increased efficiency compared to traditional transposon-mediated transgenesis [9]. Results published here, following the integration of a single phase 2 plasmid, demonstrate a proof of principle, but not an increased efficiency for site-specific integration for this particular construct. For site-specific integration, our experience with *Ae. aegypti* would indicate that size, along with other unknown characteristics of phase 2 plasmids, have a direct bearing on integration success. An inverse correlation between the size of phase 2 plasmids and transformation efficiency was also reported for site-specific integration in *D. melanogaster* [10]. The Vida3 phase 2 plasmid used here is significantly larger than the minimal plasmid used in the previous study (7.2 kb compared to 4.6 kb) [9] and the impact of expressing an effector gene is also unknown. The low efficiency reported in this study is therefore most likely due to characteristics of the phase 2 plasmid used, a hypothesis confirmed by reports of efficient integrations into our targeting strain E in unrelated projects (Nolan, *pers. comm*.). These workers injected 1571 strain E embryos to generate three independent phase 2 lines, with an estimated transformation efficiency of 10%.

Several modifications could enhance efficiency of the system in the future. Driver lines, expressing an endogenous source of integrase in the embryo from either *nanos* or *vasa* gene regulatory elements, were shown to double the transformation efficiency in *D. melanogaster* and removed the necessity to co-inject integrase mRNA [17]. These genes have now been characterised in *An. gambiae* [18], [19] facilitating the transfer of this improved technology to mosquitoes. However, *An. gambiae* is known to be difficult to transform, highlighted by the scarcity of publications [20]–[24], and efficiencies are unlikely to reach the levels reported for *D. melanogaster* or *Ae. aegypti*. Techniques have also been established for stabilisation of the initial *piggyBac* insertions by removal of one or both termini [25]–[27] should potential re-mobilisation be seen as an issue with respect to either phenotypic stability or regulatory approval. In the most recent study [27] a three-step integration and stabilisation system was designed using the phiC31 system in the Mediterranean fruit fly, *Ceratitis capitata*.

Comprehensive fitness studies involving strains E and EVida3, such as those published for *An. stephensi* [28], are ongoing and will be important for determining the likely effectiveness of such strains for potential field release. Initial studies reported here demonstrate that both fluorescent markers show normal Mendelian inheritance and that expression of the effector gene does not have a significant impact on wing length or development during normal husbandry.

Infection experiments with *P. y. nigeriensis* were consistent between replicates spanning two years, despite different levels of infection. Expression of Vida3 resulted in a significant reduction in parasite intensity compared to controls. The mean reduction in parasite burden of 79% in EVida3 is consistent with the 80% reduction in intensity previously published for the action of the peptide on *P. y. nigeriensis* during *in vitro* experiments [12]. A significant reduction in the prevalence of infection was also observed in the pooled data.

In contrast, the infection of transgenic mosquitoes with *in vitro* cultured *P. falciparum* gametocytes was more challenging. This is reflected in different relative infection rates between the two strains and inconsistency between experiments. Overall, the data suggest a trend towards reduced parasite intensity in EVida3 but no consistent significant differences between either the prevalence or intensity of infection in EVida3 compared to E. However, there is existing published evidence for an increase in *P. yoelii* infections in *An. stephensi* mosquitoes given a second uninfected bloodmeal [16] and where mosquitoes were given a subsequent bloodmeal in experiments 6 to 8, an overall significant reduction in EVida3 oocyst burden was observed. These experiments, involving multiple bloodmeals, more closely mimic the situation in the field, but conditions in the laboratory are optimised for high infections which are atypical. In addition to providing additional resources, the subsequent bloodmeal would initiate a second burst of Vida3 expression. Although we would not normally expect peptides expressed from the carboxypeptidase promoter to target oocysts developing beneath the midgut basal lamina, the Vida3 monomer presumably accessed this immune privileged site from the haemolymph in experiments where *P. berghei* oocyst numbers were significantly reduced following injection of Vida3 into the haemocoel [12]. The effect of Vida3 expression on *P. falciparum* does however appear to depend upon infection rates. We observed a significant decrease in intensity in EVida3 when control infections were high (experiments 2, 7, 8) and significant increases when control infections were low (experiments 4, 5). This is consistent with an earlier study where Yoshida *et. al*. [29] observed greater variation in the reduction of *P. falciparum* infections compared to murine parasites in transgenic *An. stephensi* expressing haemolytic C-type lectin into the midgut. Here too a significant reduction was only observed in the experiment where control infections were high. The reasons for this are unclear. Since *An. gambiae* is a natural host for *P. falciparum*, we postulate that infections are kept low by a robust and effective immune response [30]. If initial infections were high in experiments 4 and 5, an elevated immune response could be expected in strain E. A reduction in ookinete numbers by Vida3 would potentially result in reduced immune stimulation, causing resulting infections to be higher in EVida3 than E. Experiment 4 was also the only experiment where prevalence of infection was significantly increased in EVida3. A recent study of *in vitro* cultured *P. falciparum* infections reported higher parasite transmission success with increasing male sex ratios at low gametocyte densities [31]. Interestingly, the male gametocyte ratio was highest in experiment 4 (0.77 compared to 0.52 – 0.6 in the other seven experiments, Table 2) and gametocyte density was lowest (1.7×10^7^/ml, compared to 1.9 – 4.9×10^7^/ml in the other seven experiments), conditions which could favour an initially high infection in this particular experiment.

Some components of the mosquito immune response are expressed in a dose dependant manner following *Plasmodium* infection [32]. In addition, parasite developmental transitions within the mosquito are density-dependant [33]. Since *P. y. nigeriensis* but not *P. falciparum* parasites were melanised in this study, it is likely that *An. gambiae* mounts different immune responses, as is seen with *P. berghei* and *P. falciparum* infections [30], [34], [35]. These studies also identified parasite species-specific components of the mosquito immune response. The consequences of Vida3 expression on immune responses initiated by different parasites are therefore likely to be complex and highlight the importance of relevant parasite/vector choices in the future.

This study is the first report of site-specific effector gene integration and expression in *An. gambiae* and represents a significant step forward in malaria research. Vida3 consistently and significantly reduced infections of the mouse parasites against which it was originally screened, but showed inconsistent results against human malaria parasites. We are confident that ongoing work to identify peptides more active against *P. falciparum* will allow mosquitoes with significantly reduced transmission of human malaria to be generated in the near future. With this in mind, our phase 2 plasmid is modular, and designed to accept different effector genes. Moreover site-specific integration facilitates direct comparisons between alternative anti-malarial effector molecules and promoters. Ultimately the incorporation of multiple effector genes, expressed into combinations of midgut, haemocoel and salivary glands will become feasible. This is likely to be the way forward for complete transmission blockade.

## Materials and Methods

### Plasmids

pBac[3xP3-ECFPaf]-attP has been described previously [9]. The phase 2 transformation construct pBattB-DsRed2-AgCP-vida contains a 269 bp fragment flanked by *Bam*HI restriction sites and comprising four repeats of the Vida3 coding sequence ([Vida3]_4_), each separated by a flexible linker. This fragment was made by annealing two pairs of oligonucleotides (VTET 1 FOR with VTET 1 REV and VTET 2 FOR with VTET 2 REV). Annealed oligos were phosphorylated and ligated using the 4 bp overhang. Codon usage for the Vida 3 repeats in each fragment was different, to help prevent misalignment of the oligos during annealing. The fragment was digested with *Bam*HI and cloned into the *Bam*HI site of pBluescript SK+. The carboxypeptidase promoter and SM1 tetramer from pBACAgCP[SM1]_4_ [14] were removed by *Not*I digestion and cloned into pGEM-T to produce pGEM-AgCP[SM1]_4_. This was digested with *Bam*HI to replace [SM1]_4_ with the Vida3 tetramer cassette to produce pGEM-AgCP[Vida3]_4_. The AgCP[Vida3]_4_ from pGEM-AgCP[Vida3]_4_ was excised using *Not*I and cloned into the unique *Not*I site of the site-specific integration plasmid pattB-DsRed2 [9]. Microinjection DNA was prepared using the EndoFree Plasmid Maxi kit (Qiagen). Oligonucleotide sequences are given in Table S3.

### Transposase and integrase

phsp-pBac has been described previously [36]. All *attB*-*attP* integrations used phiC31 integrase mRNA transcribed from pET11phiC31poly(A) [11] using mMessage mMachine T7 Ultra (Ambion) but omitting the DNase and poly(A) tailing steps. RNA was purified (MegaClear, Ambion), precipitated and resuspended in 10–15 µl of nuclease-free water.

### Insect strains

*An. gambiae* (KIL) were maintained at 26°C±1°C and 80% RH in a 12-h light: 12-h dark photoperiod. Stock larvae were reared under standardised conditions [37] and adults fed 10% glucose *ad libitum*. Females (3–5 days old) were blood fed and preblastoderm embryos collected for microinjection 48–96 hrs post-bloodmeal. Surviving G_0_ males were backcrossed to wild-type in pools of 1–3 and females in pools of 5–10.

### Microinjection

Microinjection was performed as previously reported [9]. For phase 1, wild-type KIL strain embryos were co-injected with pBac[3xP3-ECFPaf]-attP at 333 ng/µl and phsp-pBac [36] at 200 ng/µl in 1x injection buffer and recovered without heat shock. For phase 2, strain E embryos were co-injected with pBattB-DsRed2-AgCP-vida at 250 ng/µl and phiC31 mRNA at 800 to 1000 ng/µl. Surviving G_0_ adults were backcrossed to KIL and putative G_1_ transformants identified by screening for fluorescence (Leica MZ FLIII) with filter sets from Chroma Technology (ECFP: exciter D436/20x; emitter D480/40m; DsRed: exciter HQ545/30x; emitter HQ620/60m). Transgenic strains were established from single G_1_ positive adults by backcrossing to wild-type (KIL strain).

### Infected bloodmeals

*P. y. nigeriensis* were maintained in CD1/S or TO male mice at 18°C +/− 2°C, with a 14∶10 light:dark photoperiod. Mice were initially infected with cryopreserved infected mouse blood, followed by blood passage to synchronize the infection when parasitaemia had reached approximately 10%. Infectivity was estimated by the mean number of exflagellating microgametocytes in 10 µl of mouse tail blood over 12 fields of view (x1000 magnification). Parasitaemia was assessed by Giemsa-stained thin smears from tail blood. Test and control mosquitoes (3–5 days old; glucose starved for 20 hrs) were fed simultaneously on the same mouse, followed by removal of any females not fully engorged. Mosquitoes were maintained on sugar cubes and distilled water containing 0.05% PABA without an available oviposition site. After 6–7 days, 50 midguts of each strain (when possible) were dissected with oocysts and melanised ookinetes counted at ×400 magnification. Gametocytes of *P. falciparum* NF54 isolate were produced by automated culture [38]. Details of culture conditions, assessment of maturity and preparation for feeding are as described in Mitri *et al*. [31]. Test and control mosquitoes (5 days old; glucose starved) were left to feed in the dark for 15 minutes. Fully engorged females were transferred to small cages with 10% sucrose containing 0.05% PABA. A minimum of 30 midguts were dissected after 8 days and stained with bromo-fluorescein to detect oocysts.

### Southern blotting

Genomic DNA was isolated using the Puregene DNA Purification Kit (Gentra systems) from 10 headless mosquitoes, crushed using Pellet Pestles (Anachem Ltd,), in a 5x modification to the manufacturer's *Drosophila* genomic purification protocol and resuspended in a final volume of 50 µl. Approximately 10 µg of genomic DNA was digested with *Xmn*I, *Nsi*I or *Pst*I, separated on 1% agarose and blotted onto Hybond-N+ (Amersham Biosciences). The 2195 bp probe used to detect *piggyBac* integrations was generated by *Eco*RV/*Pst*I digestion of pBac[3×P3-ECFPaf]-attP. This fragment, containing 3×P3-ECFP and a segment of the *piggyBac* left terminus was labelled with [α-^32^P]dCTP using Ready-To-Go DNA labelling beads (Amersham Biosciences). Fragment sizes were determined by comparison to the GeneRuler 1 kb DNA ladder (Fermentas UK).

### Inverse PCR

Inverse PCR was performed as described previously [9]. For strains C, G and H, the 3′ junctions were resolved using *Hae*III digestion together with primers 3′FORnew with 3′REVnew. For strain E, the 5′ junction sequence was obtained using *Taq*I digestion and primers 5′FOR with 5′REV. To resolve 5′ junctions for strains C, G and H and the 3′ junction for strain E, genomic primers were designed to the *An. gambiae* PEST sequence using primer select software (Lasergene, DNA Star). The relevant primers for strains C, G, H and E respectively were C5genomicfwd, G5genomicfwd, H5genomicfwd and E5-3R-FWD. These were used in conjunction with pBac PCR rev for 5′ junctions or 3′REVnew for the strain E 3′ junction. PCR products were cloned (pCR2.1-TOPO, Invitrogen) and sequenced (Lark Technologies). Primer sequences are given in Table S3.

### PCR analysis of site-specific integration

Unoccupied target sites were identified using primers to amplify *attP* (attR-F-new with attL-R-new) and occupied sites were identified using primers to amplify *attL* (attL-F-new-2 with attL-R-new-2) and *attR* (attR-forward with attR-reverse). 250 ng genomic DNA was amplified with the relevant primers (2 µM) using *Taq* DNA polymerase (Sigma) in 1x buffer containing 1.5 mM MgCl_2_. Cycling parameters (MJ Research PTC-100) were 94°C for 1 minute then 30 cycles (94°C, 30 seconds; 58°C, 30 seconds; 72°C, 30 seconds) followed by a final extension at 72°C for 10 minutes. PCR products were separated on 2% agarose with HyperLadder IV (Bioline). PCR products were cloned (pCR2.1-TOPO, Invitrogen) and sequenced (Lark Technologies). Primer sequences are given in Table S3.

### RT-PCR

Female mosquitoes (3 days old) were membrane fed, collected on ice at different times post-bloodmeal and frozen at −80°C. Total RNA was extracted (TRIzol, Invitrogen) and 5 µg used for reverse transcription (Superscript III, Invitrogen) with an oligo dT primer. PCRs were performed with 10% of the RT reaction as template. By limiting cycle numbers to the exponential phase (15 for *rpL7a* and 25 for Vida3) reactions were semi-quantitative and band intensity could be quantified (Bio Imaging Systems, Syngene Europe). For RT-PCR reactions, the primers AgCVIDAfwd with AgCVIDArev were used to amplify the Vida3 sequence and rpLfwd with rpLrev to amplify *rpL7a* control transcripts. PCRs were performed using *Taq* DNA polymerase (1.5U, New England BioLabs) and primers (0.5 µM, Proligo) and analysed on 1% agarose gels. Cycling parameters were as described above except that both reactions were annealed at 56°C and *rpL7a* reactions were extended for 1 minute. Primer sequences are given in Table S3.

### Statistical analysis

Mendelian inheritance and parasite prevalence were analysed by Chi-squared goodness of fit tests and wing lengths from 3 independent experiments were compared using a general linear model (Minitab). Data sets for parasite intensity were not normally distributed and were therefore analysed by Wilcoxon/Kruskal-Wallis tests applied to pairwise comparisons between the different groups of each experiment. Pooled data used a generalised linear model with Poisson distribution and overdispersion parameter (JMP7 software, e-academy Inc).

### Ethics Statement

This study was carried out in strict accordance with UK Home Office Guidelines as required by the Animals (Scientific Procedures) Act 1986 under Project Licence PPL 40/2411 (Malaria-Transmission by Mosquitoes). The protocol was approved by the Keele University Animal Ethics Committee and all efforts were made to minimize both the number of animals used and the suffering caused.

## Supporting Information

Figure S1 Parasite intensity following *P. y. nigeriensis* infections of strains E and EVida3. Oocysts and melanised ookinetes are scored as invading parasites in all experiments. Boxplots, of data from Figure 4 on the same axes, show parasite intensity for strains E (white boxes) and EVida3 (Grey boxes). Vertical lines denote the 95% confidence interval, horizontal lines with symbol mark the median value and the box marks the interquartile range of the data. Significant differences (*) for experiments are: 1, P = 0.008; 2, P = 0.0007; 3, P = 0.009; 4, P<0.0001; 5, P<0.0001 and pooled data, P<0.0001.
(0.28 MB TIF)

Table S1 Genomic DNA sequence analysis of attP integrations. Table shows strain, chromosomal location (chromosome number and arm followed by polytene map division and nucleotide number of insertion) and flanking sequences 5′ and 3′ of the piggyBac insert. All insertion sites are unique and have characteristic TTAA sequence duplications either side of the insert. The second insert site in strain C could not be resolved by inverse PCR.
(0.04 MB DOC)

Table S2 Mendelian inheritance of fluorescent markers. Hemizygous populations of strains E, G, H and EVida3 were crossed inter-se and egg batches collected from individual females. Approximately 100 F1 progeny, from a minimum of five egg batches for each strain, were screened for fluorescence. All individual F1 populations fitted the expected 3∶1 phenotypic ratio of fluorescence to wild-type. P>0.2 for all individual populations, with P for pooled data shown above (n.s. - not significant).
(0.03 MB DOC)

Table S3 Oligonucleotides and primers. Oligonucleotides VTET 1 FOR, VTET 1 REV, VTET 2 FOR and VTET 2 REV were annealed to form the Vida3 tetramer insert. Primers 3′FORnew, 3′REVnew, 5′FOR and 5′REV were used for inverse PCR and C5genomicfwd, G5genomicfwd, H5genomicfwd, E5-3R-FWD, pBac PCR rev and 3′REVnew for genomic PCR. Primers attR-F-new, attL-R-new, attL-F-new-2, attL-R-new-2, attR-forward and attR-reverse were used in PCR reactions to confirm site-specific integration. Primers AgCVIDAfwd with AgCVIDArev or rpLfwd with rpLrev were used in RT-PCR.
(0.05 MB DOC)
