# Supplementary figures and images for: Site-Specific Integration and Expression of an Anti-Malarial Gene in Transgenic Anopheles gambiae Significantly Reduces Plasmodium Infections
Source: PLoS One. 2011 Jan 25;6(1):e14587. doi: 10.1371/journal.pone.0014587 (PMC3026776; doi:10.1371/journal.pone.0014587)

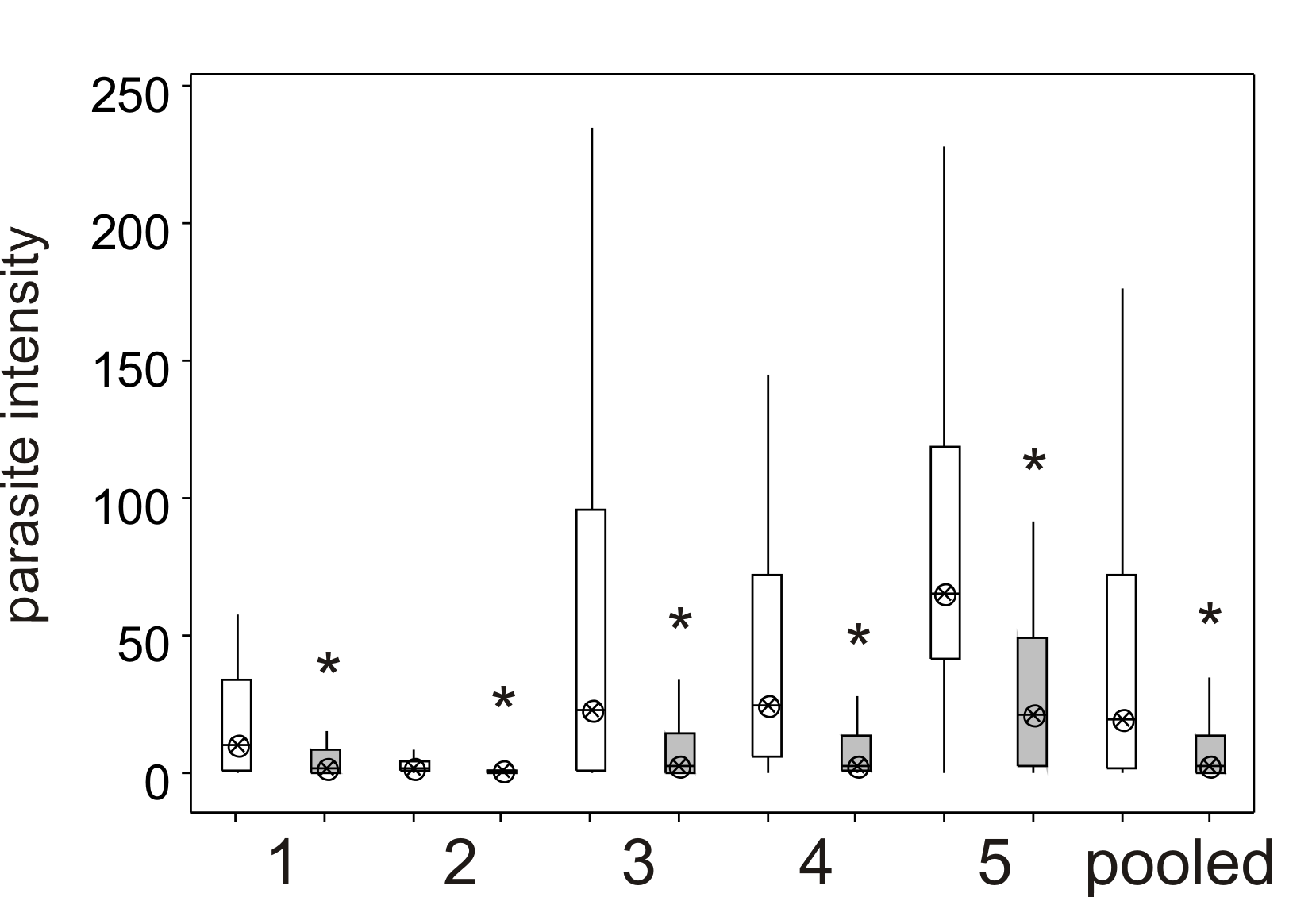

Supplement: Figure S1 — Parasite intensity following P. y. nigeriensis infections of strains E and EVida3. Oocysts and melanised ookinetes are scored as invading parasites in all experiments. Boxplots, of data from Figure 4 on the same axes, show parasite intensity for strains E (white boxes) and EVida3 (Grey boxes). Vertical lines denote the 95% confidence interval, horizontal lines with symbol mark the median value and the box marks the interquartile range of the data. Significant differences (*) for experiments are: 1, P = 0.008; 2, P = 0.0007; 3, P = 0.009; 4, P<0.0001; 5, P<0.0001 and pooled data, P<0.0001. (0.28 MB TIF) [file pone.0014587.s001.tif]
