# Supplementary material for: Site-Specific Integration and Expression of an Anti-Malarial Gene in Transgenic Anopheles gambiae Significantly Reduces Plasmodium Infections
Source: PLoS One. 2011 Jan 25;6(1):e14587. doi: 10.1371/journal.pone.0014587 (PMC3026776; doi:10.1371/journal.pone.0014587)
Supplement: Table S1 — Genomic DNA sequence analysis of attP integrations. Table shows strain, chromosomal location (chromosome number and arm followed by polytene map division and nucleotide number of insertion) and flanking sequences 5′ and 3′ of the piggyBac insert. All insertion sites are unique and have characteristic TTAA sequence duplications either side of the insert. The second insert site in strain C could not be resolved by inverse PCR. (0.04 MB DOC) [file pone.0014587.s002.doc]

**Table S1. Inverse PCR analysis of *attP* integrations.**

| **strain** | **chromosomal location** | **5’ flanking sequence** |  | **3’ flanking sequence** |
| --- | --- | --- | --- | --- |
| C | 3R 37A | ATAAAATCAACGGATAGAT | TTAA |  |
|  | 48484560 |  | TTAA | GTAAACCGCAACAACTGGC |
| E | 3R 31B | TCTCGCTAATGTAAACAGT | TTAA |  |
|  | 15801959 |  | TTAA | TTTAAGGCAACCCTTACTT |
| G | 2L 22E | ATCGCATAGGCAAATGTCA | TTAA |  |
|  | 18922078 |  | TTAA | ACGCATATCTAATAAATTT |
| H | 2L 20A | TGTAATACATGATTACTTT | TTAA |  |
|  | 1316139 |  | TTAA | ATATAGTTAAATTTTTCAA |
