# Supplementary material for: Site-Specific Integration and Expression of an Anti-Malarial Gene in Transgenic Anopheles gambiae Significantly Reduces Plasmodium Infections
Source: PLoS One. 2011 Jan 25;6(1):e14587. doi: 10.1371/journal.pone.0014587 (PMC3026776; doi:10.1371/journal.pone.0014587)
Supplement: Table S2 — Mendelian inheritance of fluorescent markers. Hemizygous populations of strains E, G, H and EVida3 were crossed inter-se and egg batches collected from individual females. Approximately 100 F1 progeny, from a minimum of five egg batches for each strain, were screened for fluorescence. All individual F1 populations fitted the expected 3∶1 phenotypic ratio of fluorescence to wild-type. P>0.2 for all individual populations, with P for pooled data shown above (n.s. - not significant). (0.03 MB DOC) [file pone.0014587.s003.doc]

**Table S2**. **Mendelian inheritance of fluorescent markers.**

| **strain** | **fluorescent** | **non-fluorescent** | **χ^2^ (1 d.f.)** | ***P*** |
| --- | --- | --- | --- | --- |
| E | 403 | 138 | 0.075 | 0.78 n.s. |
| G | 385 | 144 | 1.392 | 0.24 n.s. |
| H | 480 | 156 | 0.075 | 0.78 n.s. |
| EVida3 | 443 | 162 | 1.019 | 0.31 n.s. |
