# Supplementary material for: Site-Specific Integration and Expression of an Anti-Malarial Gene in Transgenic Anopheles gambiae Significantly Reduces Plasmodium Infections
Source: PLoS One. 2011 Jan 25;6(1):e14587. doi: 10.1371/journal.pone.0014587 (PMC3026776; doi:10.1371/journal.pone.0014587)
Supplement: Table S3 — Oligonucleotides and primers. Oligonucleotides VTET 1 FOR, VTET 1 REV, VTET 2 FOR and VTET 2 REV were annealed to form the Vida3 tetramer insert. Primers 3′FORnew, 3′REVnew, 5′FOR and 5′REV were used for inverse PCR and C5genomicfwd, G5genomicfwd, H5genomicfwd, E5-3R-FWD, pBac PCR rev and 3′REVnew for genomic PCR. Primers attR-F-new, attL-R-new, attL-F-new-2, attL-R-new-2, attR-forward and attR-reverse were used in PCR reactions to confirm site-specific integration. Primers AgCVIDAfwd with AgCVIDArev or rpLfwd with rpLrev were used in RT-PCR. (0.05 MB DOC) [file pone.0014587.s004.doc]

**Table S3. Oligonucleotides and primers.**

| **Oligonucleotide** | **Sequence** |
| --- | --- |
| VTET 1 FOR | 5’-TCCAGGACTCCCTACAAAAAGGAAAGGGATACCCCGCCGGAACTTAGGA  AATTTGCCCGGCGAGCCCACGAACAGAAACGGAATGCCTCGTCGAAATTTCG  GGAACTTGCCCGGGGATCCAC-3’ |
| VTET 1 REV | 5’-TCCAGGACTCCCTACAAAAAGGAAAGGGATACCCCGCCGGAACTTAGGA  AATTTGCCCGGCGAGCCCACGAACAGAAACGGAATGCCTCGTCGAAATTTCG  GGAACTTGCCCGGGGATCCAC-3’ |
| VTET 2 FOR | 5’-TGGAAAGTTCCCGAAATTTCGACGAGGCATTCCGTTTCTGTTCGTGGGC  TCGCCGGGCAAATTTCCTAAGTTCCGGCGGGGTATCCCTTTCCTTTTTGTAG  GGAGTCCTGGATACCCCTACGACGTGCCCGACTACGCCGGATCCCGAT-3’ |
| VTET 2 REV | 5’-CGGGATCCGGCGTAGTCGGGCACGTCGTAGGGGTATCCAGGACTCCCTA  CAAAAAGGAAAGGGATACCCCGCCGGAACTTAGGAAATTTGCCCGGCGAGCC  CACGAACAGAAACGGAATGCCTCGTCGAAATTTCGGGAACTT-3’ |
| 3’FORnew | 5’-CATTTGCCTTTCGCCTTATTTTAGA-3’ |
| 3’REVnew | 5’-AAACCTCGATATACTGACCGATAAAAACAC-3’ |
| 5’FOR | 5’-TCTTGACCTTGCCACAGAGG-3’ |
| 5’REV | 5’-TGACACTTACCGCATTGACA-3’ |
| C5genomicfwd | 5’-CGAACCACCTGAAAACGTAACACTAA-3’ |
| G5genomicfwd | 5’-GTTTTTCCACTTTTCCGTCCAATA-3’ |
| H5genomicfwd | 5’-TGTGTTTTCTAGTGTTACGGCTGTCA-3’ |
| E5-3R-FWD | 5’-TTCGGGGGTAAACAATGATAACAA-3’ |
| pBac PCR rev | 5’-ATATGCTCATCGTCTAAAGAACTACCC-3’ |
| attR-F-new | 5’-CAAATGTGTTCTGTGATGACCTG-3’ |
| attL-R-new | 5’-CTCCCTTGCTACTGACATTATGG-3’ |
| attL-F-new-2 | 5’-GAGGTCGACGATGTAGGTCAC-3’ |
| attL-R-new-2 | 5’-ACCTTTTCTCCCTTGCTACTGAC-3’ |
| attR-forward | 5’-TCAAACTAAGGCGGAGTGG-3’ |
| attR-reverse | 5’-GATGGGTGAGGTGGAGTACG-3’ |
| AgCVIDAfwd | 5’-CGGTGGCGCTCAGTGTCG-3’ |
| AgCVIDArev | 5’-CTTCAGGATCCGGCGTAGTCG-3’ |
| rpLfwd | 5’-CCCGCTGGCCAAACCGAAAAA-3’ |
| rpLrev | 5’-GTGGGGGAGGAAACGTTGGAATCT-3’ |
